# Supplementary material for: Interfering with Color Response by Porphyrin-Related Compounds in the MTT Tetrazolium-Based Colorimetric Assay
Source: Int J Mol Sci. 2022 Dec 29;24(1):562. doi: 10.3390/ijms24010562 (PMC9820508; doi:10.3390/ijms24010562)
Supplement: Supplementary file 1 [file ijms-24-00562-s001.zip › ijms-2095719-supplementary.pdf]

Supplementary Figure Caption

Supplementary Figure S1. Structures of zinc protoporphyrin IX (ZnPP, A), protoporphyrin IX disodium salt (PPIX, B), zinc phthalocyanine (ZnPC, C), cyanocobalamin (CBL, D), and hemin (E) used in the present study.

Supplementary Figure S2. Photos of INT 407 cell morphology after 24 h treatment of ZnPP, PPIX and ZnPC for 24 h (each 10 μM)

Supplementary Figure S3. Effect of Zn on stability of MTT formazan under light. MTT formazan formed by cells was incubated without or with different concentrations of Zn under light at room temperature for 4 h. Each value represents the mean±S.D. (n=4). n.s., not significant.

Supplementary Figure S4. Effects of porphyrins on degradation of water soluble formazans including XTT (A), MTS (B), WST-1 (C) and WST-8 (D). The water soluble formazans formed from different tetrazolium were mixed with each porphyrin, and the mixture was incubated at room temperature under light condition. Changes of color response were analyzed during 3 h. Each value represents the mean±SD (n=3). *, ** Significantly different from initial control according to Student's t-test (*, $p<0.05$; **, $p<0.01$).

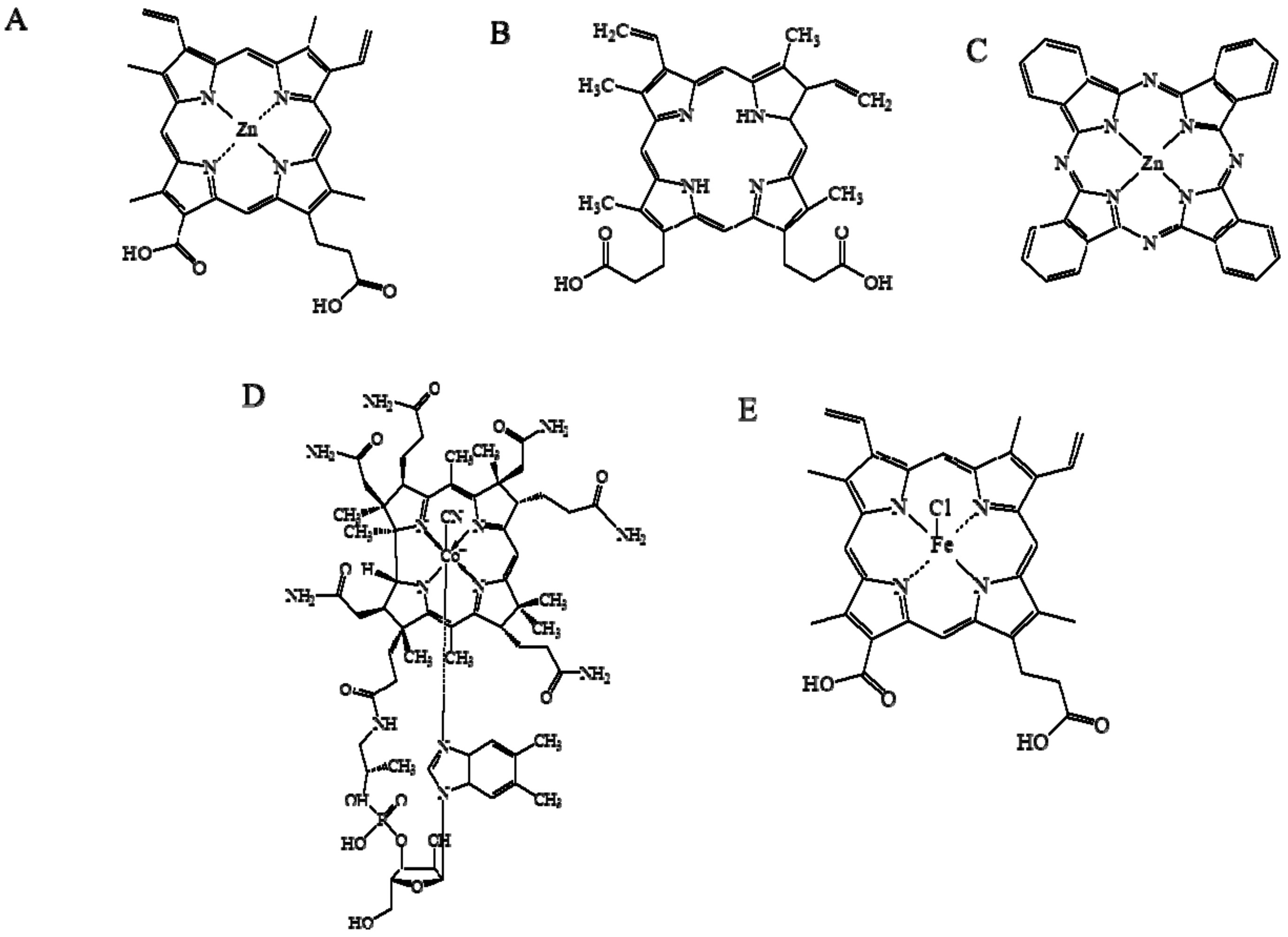

Supplementary Figure S1

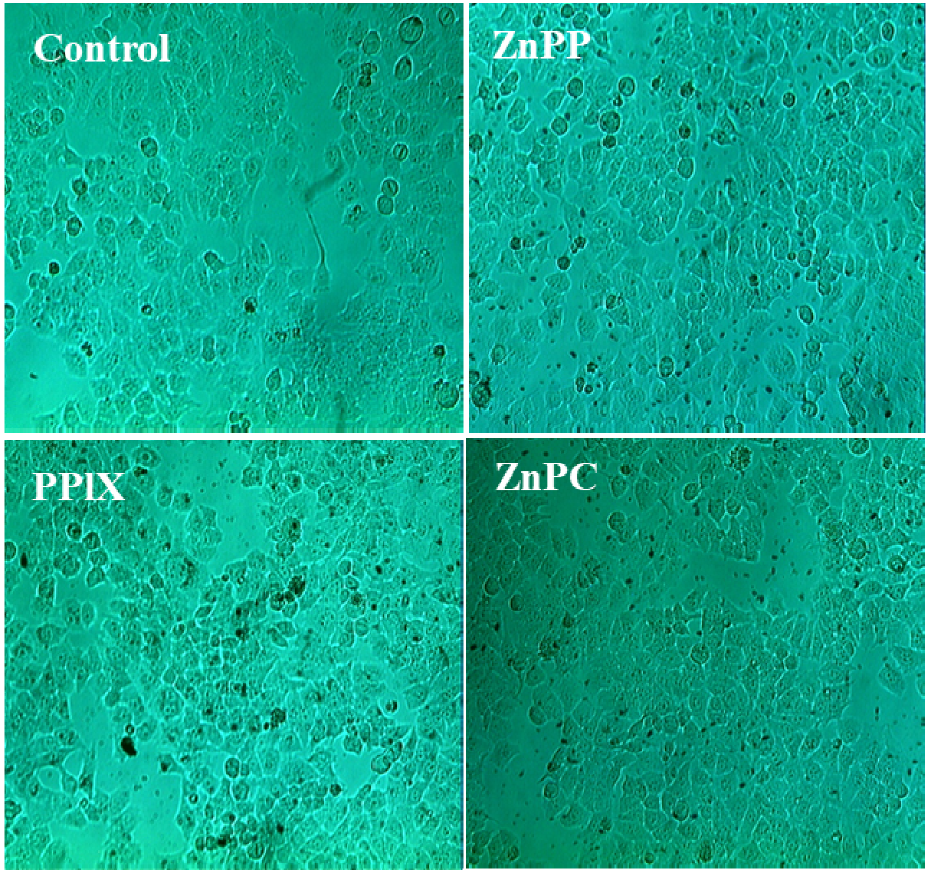

Supplementary Figure S2

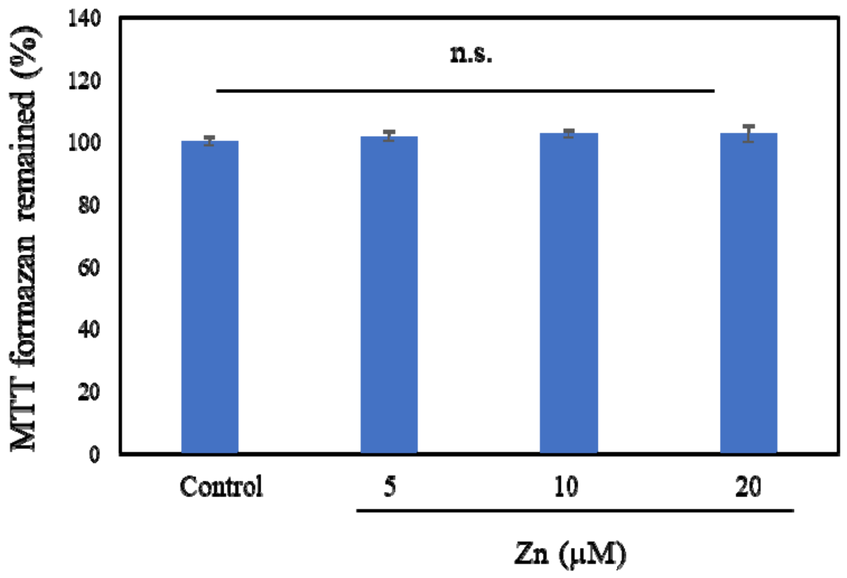

Supplementary Figure S3

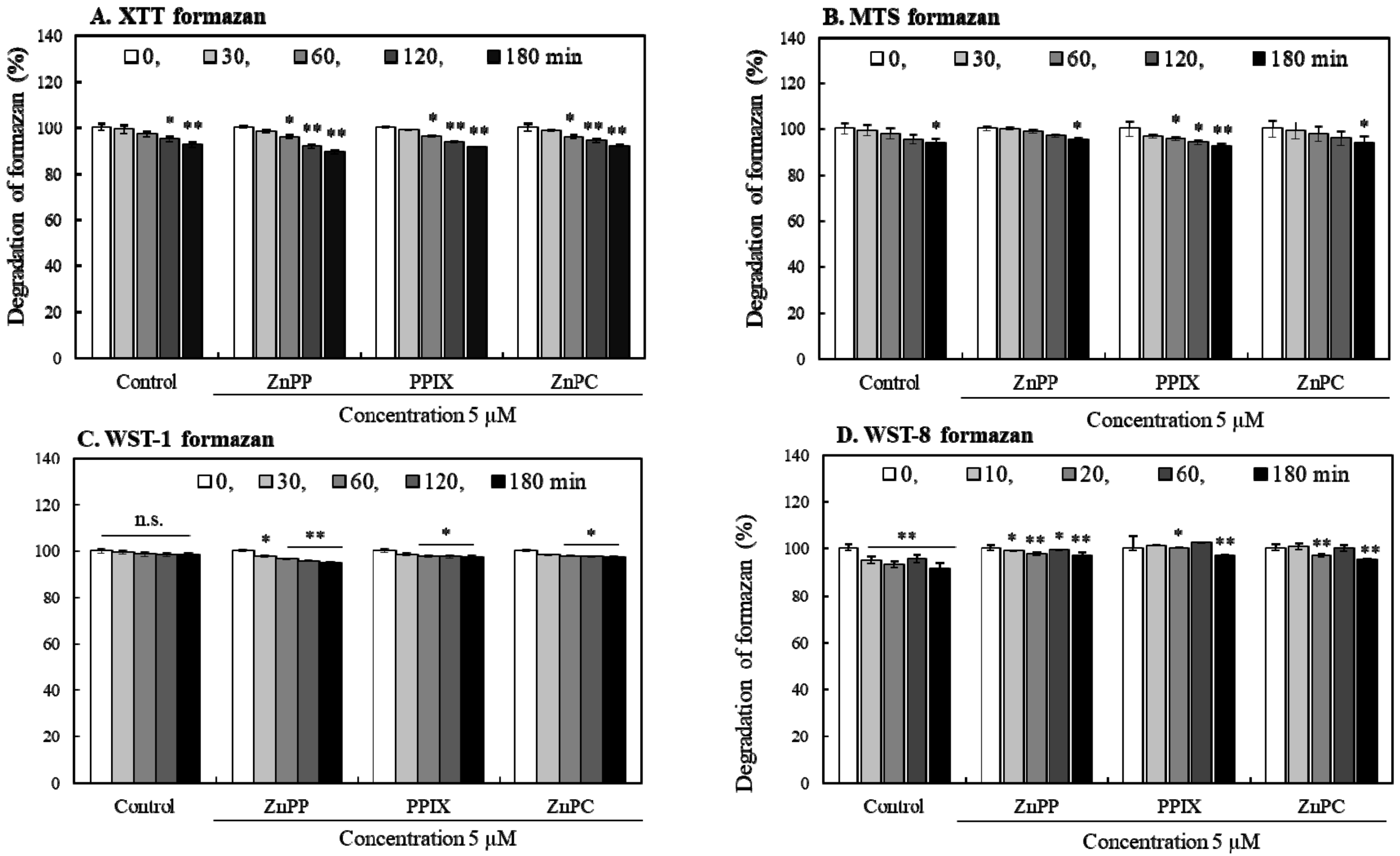

Supplementary Figure S4
